# Biomarkers of Environmental Pollutants

**DOI:** 10.1155/2014/806598

**Published:** 2014-12-22

**Authors:** Anilava Kaviraj, Erhan Unlu, Abhik Gupta, Ahmed El Nemr

**Affiliations:** ^1^Department of Zoology, University of Kalyani, Kalyani, West Bengal 741235, India; ^2^Department of Biology, University of Dicle, 21280 Diyarbakir, Turkey; ^3^School of Environmental Sciences, Assam University, Silchar 788011, India; ^4^Marine Pollution Department, National Institute of Oceanography and Fisheries, Kayet Bey, Elanfoushy, Alexandria 21556, Egypt

Thousands of pollutants enter daily into environment and exert various kinds of stress on organisms and ecosystems. Risk assessment of these pollutants to organisms and ecosystems is challenging because of diversities in chemical nature and mode of toxicity of the pollutants as well as variation in sensitivities of the organisms exposed to the pollutants. Even low, relatively innocuous concentrations of pollutants often produce deleterious effects on organisms, which are difficult to be predicted, because measurable effects are expressed only after prolonged exposure. But it may be too late to take remedial actions or steps to reduce risk when these incipient effects are expressed. Therefore, it is necessary to develop early warning signals or biomarkers that convincingly reflect adverse biological responses towards anthropogenic environmental toxins even at minute concentrations [1]. A biomarker is defined as “a change in biological response, ranging from molecular through cellular and physiological responses to behavioral changes, which can be related to exposure to or toxic effects of environmental chemicals” [2]. Recent developments in molecular biology and biotechnology and inventions of sophisticated instruments have led to the development of novel, more sensitive validated biomarkers of exposure, effect, and susceptibility to the adverse effects of terrestrial and aquatic pollutants [3]. Validity of these biomarkers has been tested in several laboratory and field studies [1]. In addition, systematic use of multiple biomarkers has been found as most useful in the assessment of pollutants' effects [4].

This special issue on biomarkers of environmental pollutants contains ten papers, which discussed some recent applications of biomarkers in terrestrial and aquatic environment. A few papers deal with a single sensitive biomarker, while others deal with combinations of selected biomarkers. The papers have been categorized under three main groups.

*Biomarkers of Susceptibility/Oxidative Biomarkers*. Two papers have been published in this category. One paper deals with perfluorooctanoic acid (PFOA), which is widely present in the environment. Oxidative stress induced by PFOA on mice was evaluated by malondialdehyde (MDA) formation and hydrogen peroxide generation, which were considered as reliable biomarkers of anthropogenic stresses on mice. The second paper in this category also deals with MDA. Elevation in MDA associated with depletion in antioxidant enzymes activities in the tissues was measured in liver and kidney of female rats exposed to lead acetate. The authors observed that Omega-3 polyunsaturated fatty acid provided a protection against lead acetate toxicity in rats.

*Biomarkers of Exposure*. Four papers have been published in this category. In one paper the authors established that deposition of mercury in hair served as an excellent biomarker of exposure of human population to mercury. In another paper differences in whole blood versus tibia lead concentrations over time in growing rats were evaluated prenatally and higher amount of lead found in the bone of older animals reinforced the importance of using bone lead as an exposure biomarker. In the third paper it was established that coelomic fluid instead of whole earthworm could be used to identify ecologically relevant end points. Induction of metallothionein in the coelomic fluid of earthworms served as sensitive biomarkers of heavy metal pollution in soil. The fourth paper discussed application of multiple biomarkers to assess risk of corals. Several biomarkers enzymes involved in melanin synthesis pathway (phenoloxidase (PO) and peroxidases (POD)) and free radical scavenging enzymes (super oxide dismutase (SOD), catalase (CAT)) and glutathione peroxidase (Gpx) were determined in selected scleractinian corals to evaluate stress induced by coral pathogen and to predict health and future existence of corals.

*Biomarkers of DNA Damage.* Two papers have been published in this category. One deals with effects of contaminants on DNA damages of Zebra Mussels* Dreissena polymorpha* evaluated by Comet assay and micronucleus test (MNT) and another on DNA damage and telomerase activity in exfoliated urinary cells of human workers of rubber tire industry evaluated by Comet assay and quantitative telomerase repeat amplification protocol (TRAP) and fluorescence in situ hybridization (FISH) assay. MNT appeared as better tool than Comet assay in Zebra mussel while TRAP, Comet, and FISH assays worked well as early biomarkers of procarcinogenic effects on rubber factory workers.

*Reviews on Biomarkers.* Two review papers have been published. One review paper discussed variations in the expression levels of heat shock protein (HSP) under a variety of toxic conditions. The paper discussed current knowledge on expression of HSPs and concluded that HSP was unreliable as biomarker due to synergistic effects of toxicants and other environmental factors. Another review paper deals with biomarkers of type-II synthetic pyrethroid pesticides. Applications of hematological, enzymatic, cytological, genetic, omic, and other types of biomarkers to evaluate toxicity of the major type-II pyrethroid pesticides in freshwater fish species have been documented. The papers discussed the present status of researches on type-II pyrethroids and the possible future directions.

It is concluded that till today oxidative biomarkers are the most frequently used category of biomarkers to assess adverse effects of a wide variety of pollutants. In the quest to develop more specific biomarkers researchers are concentrating on emerging areas with metabolomics and proteomics forming the frontier area of biomarker research. These trends are being facilitated by the development of increasingly precise and sensitive instrumentation in recent years.

*Anilava  Kaviraj* *Erhan  Unlu* *Abhik  Gupta* *Ahmed  El  Nemr*
